# Ultra-low-dose in brain 18F-FDG PET/MRI in clinical settings

**DOI:** 10.1038/s41598-022-18029-7

**Published:** 2022-09-12

**Authors:** Marine Soret, Jacques-Antoine Maisonobe, Serge Desarnaud, Sébastien Bergeret, Valérie Causse-Lemercier, Arnaud Berenbaum, Laura Rozenblum, Marie-Odile Habert, Aurélie Kas

**Affiliations:** 1 AP-HP Sorbonne Université, Hôpital Pitié-Salpêtrière, Médecine Nucléaire, 75013 Paris, France; 2 Laboratoire d’Imagerie Biomédicale, LIB, Sorbonne Université, CNRS, INSERM, 75006 Paris, France; 3 Centre d’Acquisition et Traitement des Images (CATI), Saclay, France

**Keywords:** Molecular medicine, Diagnostic markers, Neurological disorders

## Abstract

We previously showed that the injected activity could be reduced to 1 MBq/kg without significantly degrading image quality for the exploration of neurocognitive disorders in 18F-FDG-PET/MRI. We now hypothesized that injected activity could be reduced ten-fold. We simulated a 18F-FDG-PET/MRI ultra-low-dose protocol (0.2 MBq/Kg, PET_ULD_) and compared it to our reference protocol (2 MBq/Kg, PET_STD_) in 50 patients with cognitive impairment. We tested the reproducibility between PET_ULD_ and PET_STD_ using SUVratios measurements. We also assessed the impact of PET_ULD_ for between-group comparisons and for visual analysis performed by three physicians. The intra-operator agreement between visual assessment of PET_STD_ and PET_ULD_ in patients with severe anomalies was substantial to almost perfect (kappa > 0.79). For patients with normal metabolism or moderate hypometabolism however, it was only moderate to substantial (kappa > 0.53). SUV ratios were strongly reproducible (SUVratio difference ± SD = 0.09 ± 0.08). Between-group comparisons yielded very similar results using either PET_ULD_ or PET_STD_. 18F-FDG activity may be reduced to 0.2 MBq/Kg without compromising quantitative measurements. The visual interpretation was reproducible between ultra-low-dose and standard protocol for patients with severe hypometabolism, but less so for those with moderate hypometabolism. These results suggest that a low-dose protocol (1 MBq/Kg) should be preferred in the context of neurodegenerative disease diagnosis.

## Introduction

In the last five years, PET technology has improved significantly with the development of new silicon-based detector technology, combined with an increased axial field of views resulting overall in higher sensitivity of modern PET cameras. For brain 18F-FDG PET/MRI, total acquisition time reduction is limited by MR, which usually lasts 20 min or more. Hence, further 18F-FDG radiotracer dose reduction may be achieved by increasing the PET acquisition duration to match that of MR^1^. We previously showed in 100 patients with suspected neurodegenerative dementia that the injected activity of 18F-FDG could be reduced by half without qualitatively or quantitatively degrading 18F-FDG PET/MR image quality^2^. Other studies have used sophisticated deep learning approaches to produce high-quality PET images from low-dose PET acquisitions, or to denoise low-dose PET acquisitions^3,4^. These methods enable a substantial dose reduction without noise increase or loss in image quality, but have mostly been explored in research applications and have yet to become widely accessible in the clinical setting.

In the present study, we sought to assess whether the injected activity of 18F-FDG could be further decreased in the clinical setting to as low as ten-fold less than current recommendations for brain imaging, by reducing scanning time in clinical settings. We also evaluated the reliability of images resulting from this ultra-low-dose 18F-FDG PET/MR protocol in two patient groups with suspected neurodegenerative dementia, the first with normal metabolism or moderately decreased metabolism, and the second with severe hypometabolism.

## Material and methods

We retrospectively simulated the stepwise reduction of 18F-FDG injected activity in 50 patients (67.4 ± 7.2 years old, 26 men) who underwent 18F-FDG PET on a SIGNA 3 T PET/MR system (GE Healthcare) for cognitive impairment or suspected neurodegenerative dementia. These patients had an average weight of 70 ± 11 kg and pre-scan blood glucose level ranging from 5 to 8 mmol/L.

The raw data were extracted from a previously described cohort of patients^2^ belonging to the Pitié-Salpêtrière Hospital database. This database was approved by the French authority for the protection of privacy and personal data in clinical research (CNIL, approval No. 2111722). The use of patient data was performed in accordance with French regulations on bioethics and medical research. In France, according to the law “loi Jardé” (article L. 1121-1 of the public health code) governing scientific research on human subjects, patient data can be used for retrospective scientific research without the prior approval of an ethical committee. All subjects had given their consent for the use of their data for research purposes. We performed this study in accordance with the principles of the Declaration of Helsinki.

Following the revised EANM guidelines for 18F-FDG brain PET^5^, all scans were performed in standardized resting (reduced ambient noise, from 10 min before injection to 25 min post injection) and euglycemic conditions (at least 4 h of fasting). A 18F-FDG activity of 2 MBq/Kg was injected 31 ± 5 min prior to a 30 min brain PET/MRI including MRI sequences and a 20-min single-bed-position PET acquisition acquired simultaneously (i.e. during 18F-FDG equilibrium). 18F-FDG syringes were prepared by a Unidose automatic radiopharmaceutical dispenser (Trasis, Belgium), ranging from 125 to 250 MBq. The average injected activity per subject was 140 ± 17 MBq (between 110 and 201 MBq). PET data were reconstructed with our usual parameters used in clinical practice (OSEM-3D, 28 subsets, 8 iterations, no axial filtering, 3 mm transaxial post-reconstruction Gaussian filter) to generate a 20-min summed image, used as a reference (PET_STD_). To simulate a 0.2 MBq/Kg injected activity, a second set of images (PET_ULD_) was reconstructed over the first 2 min from list-mode data (corresponding exactly to 0.21 MBq/Kg taking into account radioactive decline). Reconstruction parameters of PET_ULD_ were optimized by physicists to ensure a high signal to noise ratio, for instance by increasing filtering (OSEM-3D, 32 subsets, 6 iterations, light axial filtering, 5 mm transaxial post-reconstruction Gaussian filter). The method to simulate low-dose datasets has been previously validated with experiments conducted with a Hoffman 3D brain phantom (BR/3D/P model, Data Spectrum Corporation) and has been extensively described in Soret et al.^2^

We first evaluated the impact of an ultra-low-dose protocol on PET visual interpretation by evaluating intra-operator reproducibility. All PET volumes were reoriented along the bicommissural line. For visual interpretation, PET volumes were displayed in three orthogonal views with the French color scale. The minimal SUV value was set at 0 and the maximum was defined on midbrain uptake, for intensity normalization. Reading was done using an Advantage Workstation of GE Healthcare. Three raters with more than 10 years’ experience analyzed PET_STD_ and PET_ULD_ images independently, and classified 18F-FDG PET patterns into seven categories: normal (N), non-evaluable (NE), Alzheimer’s disease (AD), logopenic variant progressive primary aphasia (lvPPA), posterior cortical atrophy (PCA), semantic variant progressive primary aphasia (svPPA), or frontotemporal dementia (FTD). The reading sessions for PET_STD_ and PET_LD_ were separated by more than 3 months. The available clinical information during reading sessions were only the age and sex of the patients, one or more suspected clinical diagnoses, or the prominent symptoms at presentation. We assessed the intra-operator agreement with Cohen’s kappa tests with 95% confidence intervals (CI), and the degree of agreement was characterized by kappa magnitude as defined by Landis and Koch^6^. At the end of the two reading sessions, a consensus on the final imaging diagnosis was reached among the three raters, based on the PET_STD_ images.

We then extracted cortex-to-pons uptake ratios (SUVR) from PET_STD_ (SUVR_STD_) and PET_ULD_ (SUVR_ULD_) in 20 regions-of-interest with CortexID Suite software (v2.1, General Electric Healthcare), compared them with a paired Student’s T-test, and calculated the bias as ΔSUVR = |SUVR_STD_ – SUVR_ULD_|. We assessed the reproducibility between SUVR measurements with a Bland–Altman graphical analysis. To evaluate the impact of the ultra-low-dose protocol according to the severity of 18F-FDG anomalies, we formed two sub-groups: (1) patients with “severe hypometabolism” as defined by PET_STD_ quantification revealing at least 3 hypometabolic regions with a Z-score ≤ -3 relative to the CortexID normal database; (2) patients with “normal to mildly decreased metabolism” comprising of the remaining patients.

Lastly, we assessed the impact of using PET_ULD_ rather than PET_STD_ on the results of between-group comparisons (AD versus FTD patients) using a VOI analysis as previously described^2^.

## Results

Based on the consensus reading between the three readers, PET_STD_ was visually normal in 16 patients and suggestive of AD in 15, svPPA in 6, FTD in 8 and lvPPA or PCA in 5 (Table 1). Based on PET_STD_ quantitative analysis, 20 patients were classified in the category “severe hypometabolism”. Thirty patients were classified as having “normal PET or moderate hypometabolism” (Table 1).

**Table 1:** Number of patients classified for each metabolic pattern according to consensus based on PET_STD_: patients were also classified in two categories “severe hypometabolism” and “normal/moderate hypometabolism” according to regional PET_STD_ quantification.

Metabolic pattern	AD	lvPPA	PCA	svPPA	FTD	N	NE	All
With severe hypometabolism (66.0 ± 5.7 year, 12 males)	7	2	2	2	7	0	0	20
With normal/moderate hypometabolism (68.4 ± 7.2 year, 14 males)	8	1	0	4	1	16	0	30
All patients (67.4 ± 7.2 year, 26 males)	15	3	2	6	8	16	0	50

*N* Normal, *NE* non-evaluable, *AD* Alzheimer’s disease, *lvPPA* logogenic variant of progressive primary aphasia, *PCA* posterior cortical atrophy, *svPPA* semantic variant of progressive primary aphasia, *FTD* frontotemporal dementia.

**Figure 1:**
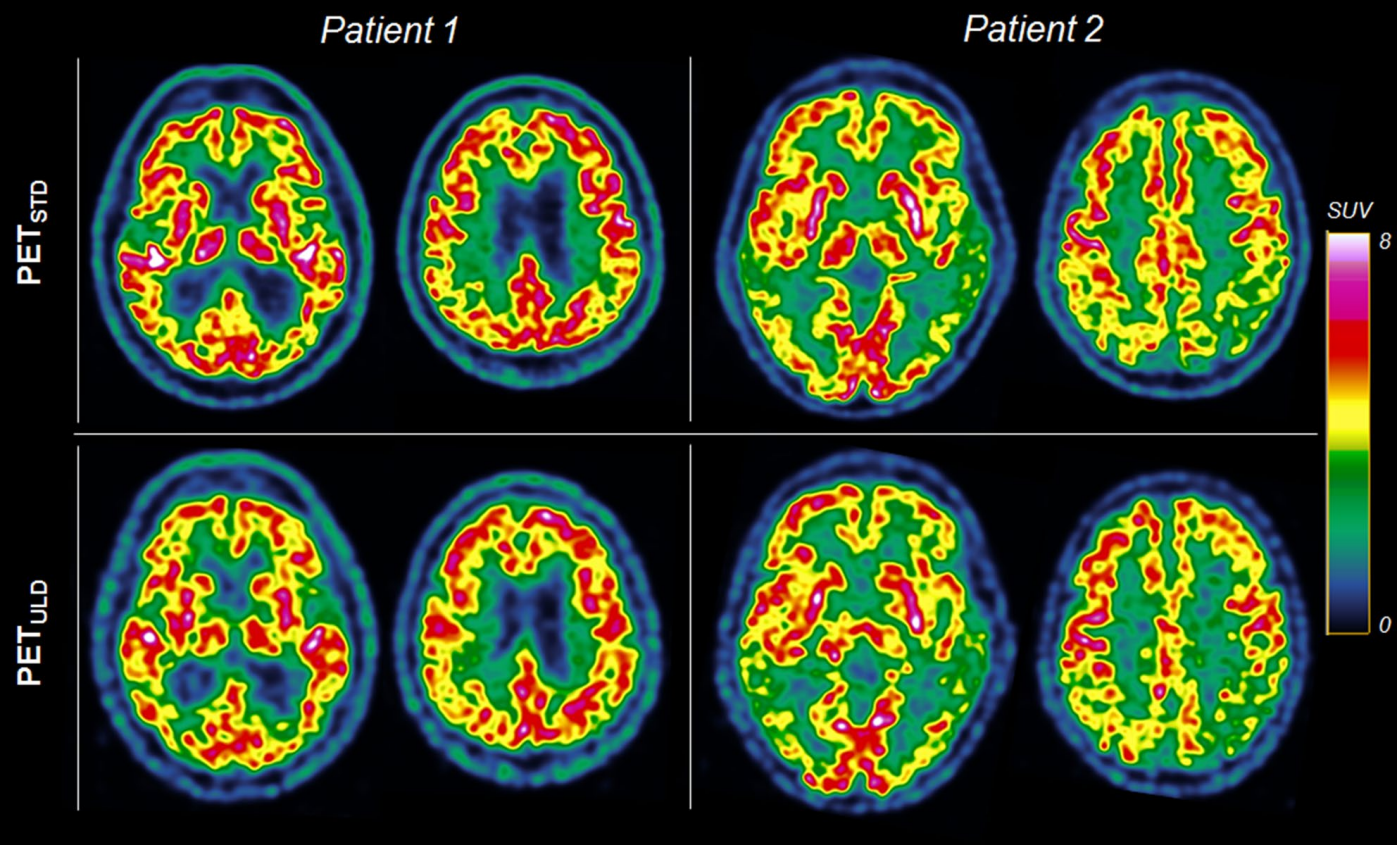
Transverse slices of two patients suspected of AD with mild hypometabolism (patient 1) and severe hypometabolism (patient 2) with standard dose (PET_STD_, 2 MBq/kg) and ultra-low dose (PET_ULD,_ 0.2 MBq/kg) protocol. The transverse slices are extracted from the Advantage Workstation (AW server 3.2, GE Healthcare) used to carry out the interpretation.

Figure 1 shows transverse slices of two patients suspected of AD obtained with standard and ultra-low dose protocol.

Intra-operator agreement between PET_STD_ and PET_ULD_ for visually classifying patients into seven categories (Table 2) was almost perfect in one rater (Cohen’s kappa 0.95) and substantial in the two others (≥ 0.71) over the whole group, whereas in the 20 patient sub-group with severe hypometabolism it was almost perfect for 2 raters (Cohen’s kappa ≥ 0.86) and substantial for the other (0.79).

**Table 2:** Intra-operator concordance between PET_STD_ and PET_ULD_ visual analyses.

Patient groups	Reader #1	Reader #2	Reader #3
With severe hypometabolism (n = 20, age 66.0 ± 5.7 year, 12 males)	1.00 (1.0–1.0)	0.86 (0.67–1)	0.79 (0.59–1)
With normal/moderate hypometabolism (n = 30, 68.4 ± 7.6 year , 14 males)	0.85 (0.68–1.0)	0.53 (0.27–0.79)	0.62 (0.39–0.84)
All patients (n = 50, 67.4 ± 7.2 year, 26 males)	0.95 (0.87–1)	0.76 (0.61–0.90)	0.71 (0.55–0.86)

Cohen’s kappa value and 95% confidence interval characterized by (lower limit-upper limit) are shown. An intra-operator concordance of 1 means that the classification into 7 categories (N, NE, AD, lvPPA, PCA, svPPA, FTD) is the same when the reader visually interprets PET_STD_ and PET_ULD._ Landis and Koch arbitrary characterized kappa values 0–0.20 as slight agreement, 0.21–0.40 as fair, 0.41–0.60 as moderate, 0.61–0.80 as substantial, and 0.81–1 as almost perfect agreement^6^.

In this latter sub-group, the diagnosis based on PET_STD_ was reconsidered on PET_ULD_ in 0/20, 2/20, 3/20 patients by reader #1, 2, 3, respectively. However, when considering patients with normal/moderate hypometabolism, agreement between PET_STD_ and PET_ULD_ was lower (moderate for one rater and substantial for the 2 others, Cohen’s kappa ≥ 0.53), and the diagnosis was reconsidered in 3/30, 9/30, and 8/30 patients for reader #1, 2, and 3, respectively (Table 2).

**Figure 2:**
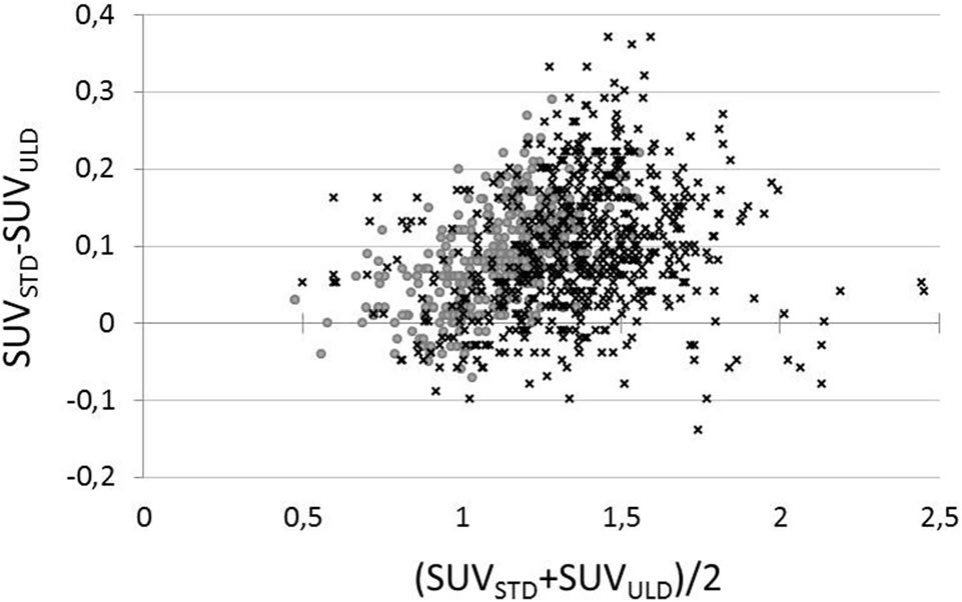
Bland–Altman plot of regional differences SUVR_STD_ – SUVR_ULD_ as a function of (SUVR_STD_ + SUVR_ULD_)/2 from all 20 VOIs for patients with normal/moderate hypometabolism (×) and patients with severe hypometabolism (·).

SUVR_ULD_ and SUVR_STD_ measured over the 20 VOIs were statistically different (p < 0.05) (Table 3). The quantitative bias between regional SUVR_STD_ and SUVR_ULD_ measurements was negligible (mean ΔSUVR ± SD:0.09 ± 0.08, 95% limits of agreement [− 0.07; − 0.24], Fig. 2) with the greatest differences involving the right (12.1%) and left (11.5%) posterior cingulate.

**Table 3:** Regional SUVR_ULD,_ SUVR_STD_ and difference ΔSUVR% = (SUVR_STD_ – SUVR_ULD_)/SUVR_STD_ expressed as mean ± standard deviation.

	Patients with severe hypometabolism			Patients with normal/moderate hypometabolism			All patients		
	SUVR_STD_	SUVR_ULD_	ΔSUVR %	SUVR_STD_	SUVR_ULD_	ΔSUVR %	SUVR_STD_	SUVR_ULD_	ΔSUVR %
Left ant. cingulate	1.12	1.03	8.5*	1.21	1.13	6.2*	1.17	1.08	7.1*
Right ant. cingulate	1.07	0.99	7.6*	1.20	1.13	6.5*	1.15	1.07	6.9*
Left occipital lateral	1.30	1.20	7.8*	1.59	1.46	8.5*	1.48	1.36	8.3*
Right occipital lateral	1.28	1.19	7.0*	1.60	1.49	6.9*	1.47	1.37	7.3*
Left parietal inf	1.15	1.07	7.0*	1.49	1.38	7.2*	1.35	1.26	7.1*
Right parietal inf	1.14	1.06	7.4*	1.49	1.39	6.6*	1.35	1.26	6.8*
Left parietal sup	1.18	1.06	10.4*	1.46	1.31	9.8*	1.35	1.21	10.0*
Right parietal sup	1.13	1.02	9.0*	1.45	1.34	7.0*	1.32	1.22	7.6*
Left post. cingulate	1.24	1.10	11.7*	1.66	1.47	11.4*	1.50	1.32	11.5*
Right post. cingulate	1.24	1.09	12.2*	1.68	1.48	12.1*	1.51	1.33	12.1*
Left precuneus	1.25	1.11	11.3*	1.62	1.44	11.0*	1.47	1.31	11.1*
Right precuneus	1.22	1.11	9.0*	1.61	1.47	8.8*	1.46	1.33	8.8*
Left prefrontal lateral	1.31	1.20	8.8*	1.52	1.41	7.4*	1.43	1.32	7.9*
Right prefrontal lateral	1.27	1.18	6.7*	1.50	1.43	4.9*	1.41	1.33	5.6*
Left prefrontal medial	1.24	1.14	8.1*	1.38	1.30	6.1*	1.32	1.23	6.8*
Right prefrontal medial	1.18	1.11	6.0*	1.35	1.28	5.1*	1.28	1.21	5.4*
Left temporal lateral	1.11	1.07	3.4*	1.29	1.26	2.7*	1.21	1.18	2.9*
Right temporal lateral	1.06	1.04	2.2*	1.31	1.28	1.6*	1.21	1.19	1.8
Left temporal mesial	0.95	0.96	− 1.1	1.00	1.01	− 1.7*	0.98	0.99	− 1.4
Right temporal mesial	0.93	0.92	1.1	1.02	1.03	− 0.7	0.99	0.99	− 0.1
All	1.16 ± 0.21	1.08 ± 0.17	6.8 ± 3.7*	1.40 ± 0.29	1.31 ± 0.24	6.1 ± 3.8*	1.31 ± 0.29	1.22 ± 0.25	6.4 ± 3.8*

*Indicates statistically significant difference between SUVR_STD_ and SUVR_ULD_ with p < 0.05.

*Ant.* anterior, *Post.* posterior, *Sup.* superior, *Inf.* inferior.

The amplitude of bias was similar in patients with normal metabolism/moderate hypometabolism and, those with severe anomalies, i.e. 0.09 ± 0.062 and 0.08 ± 0.046, respectively (Table 3).

**Figure 3:**
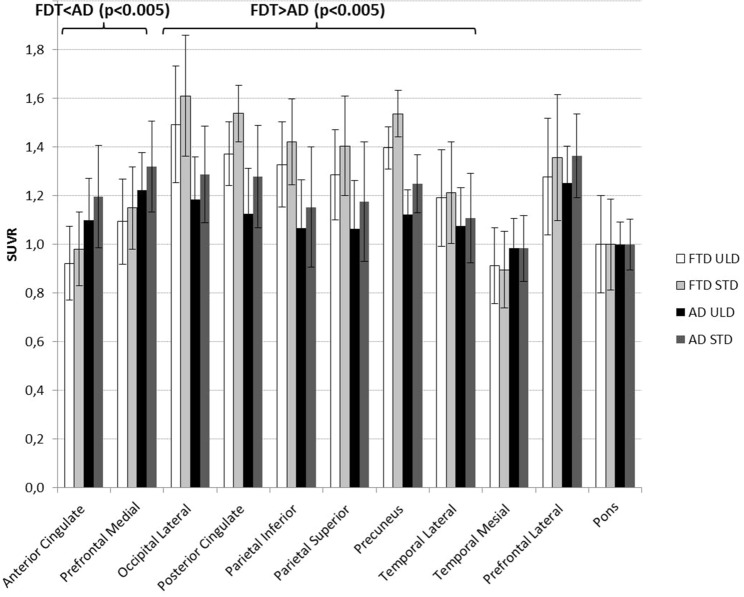
Mean and range SUVR for PET_ULD_ and PET_STD_ measured for all VOIs for patients considered as AD (15 patients) and FTD (8 patients).

Between-group comparisons (15 AD vs. 8 FTD) performed successively with PET_STD_ and PET_ULD_ generated very similar results (Fig. 3). As expected, SUVR_STD_ were significantly decreased in AD compared with FTD in the parietal, precuneus, and lateral occipital cortices (p < 0.005). VOI analysis performed with SUVR_ULD_ provided similar statistically significant results.

## Discussion

We show that performing a 20-min brain PET/MRI with a 18F-FDG activity reduced to as low as 0.2 MBq/Kg is feasible without compromising quantitative measurements. It does not change either imaging diagnosis in patients with clear regional anomalies. However, it might influence readers’ final diagnosis in equivocal profiles with sparse, mild to moderate anomalies. Our finding suggest that the ultra-low-dose protocol could be an alternative for the differential diagnosis or follow-up in patients with severe cognitive impairment or dementia. Injecting 1–2 MBq/Kg 18F-FDG should be preferred for the early diagnosis of neurodegenerative disorders to guarantee a high image quality and better diagnostic performances.

In a previous study involving 100 brain 18F-FDG PET/MRI examinations, we found that the injected activity of 18F-FDG could be reduced from 2 to 1 MBq/Kg without modifying diagnostic performances and quantitative assessments in patients with cognitive impairment^2^. In the present study, we tested a ten-fold activity reduction compared to the recommended activity (i.e. 0.2 vs. 2 MBq/Kg) resulting in a mean SUVR bias smaller than 7% over the whole cortex. The mean SUVR bias was greatest in the right posterior cingulate region (12.1%). The amplitude of bias was similar for patients with high or mild brain hypometabolism. Between-group comparisons based on SUVR_ULD_ and SUVR_STD_ similarly differentiated AD from FTD. However, the visual analysis was substantially impacted in the patient sub-group with mild metabolism change. In this sub-group, experts reconsidered the final imaging-based diagnosis when switching from the standard to the ultra-low-dose PET images in 33% of cases, probably due to uncertainty related to lower image quality. As expected, the diagnosis was reconsidered less frequently (only 8% of cases) in the group with severe hypometabolic patterns.

Our findings are consistent with those of Fällmar et al.^7,8^ who reported a minimal impact of a four-fold decrease in injected activity on SUV measurements and diagnostic information analyzed with Z-score maps in 17 brain 18F-FDG PET/CT studies (neurodegenerative disorders vs controls). Our results involving 50 subjects suggest that an even greater dose reduction may be achievable for the purpose of quantitative measurements, probably because of the greater detector sensitivity of our PET/MRI camera.

Another recent study by Schiller et al. concerned dose reduction for brain 18F-FDG PET/CT in 25 patients diagnosed with AD or FTD^9^. The authors performed VOI-based as well as voxel-based analyses, along with single-subject visual readings. All their analyses revealed the potential to divide by 4 the injected activity (from 200 to 50 MBq) without compromising diagnostic quality. In the VOI-based analysis, despite a decreased injected activity from 200 MBq down to 20 MBq, significant differences in mean normalized uptake between AD and FTD patients remained in the posterior cingulate, precuneus, temporal and parietal cortical VOIs. Our findings are in line with these results, showing that with 18F-FDG PET/MR, AD and FTD metabolic patterns can still be distinguished by VOI-based analysis even after a ten-fold decrease in injected activity. Schiller et al. also highlighted that concerning single-subject visual readings, a four-fold activity reduction is the limit in order to maintain optimal diagnostic performances in discriminating between AD and FTD^9^. However, the ability to visually detect mild pathological changes was not evaluated. Our results showed substantial agreement between standard and ultra-low-dose protocols in patients with severe hypometabolism (kappa > 0.79). However, the ultra-low-dose protocol reached its limit in patients with mild metabolic changes. As previously published, a low dose (and not ultra-low dose) protocol (1 MBq/Kg) should be preferred for visually detecting mild metabolic changes^2^.

In our study, we simulated the activity reduction by decreasing the PET duration, assuming that counting statistics would be decreased by the same factor. An alternative would have been to simulate a dose reduction by randomized subsampling of PET list-mode data^10^. However, our approach was previously validated for our clinical brain FDG PET protocol with brain phantom measurements^2^. To ensure that the comparison between 20 and 2-min PET would not be biased by metabolic changes over time, all images were strictly acquired at equilibrium and head motion was prevented by foam restraints within the head coil.

As ultra-low-dose PET typically results in images with a lower signal-to-noise ratio, some studies have used machine learning-based methods to produce diagnostic images from amyloid PET/MRI with only 1% of standard radiotracer activity^3^ or from 18F-FDG brain PET/CT with only 30% and 10% of standard radiotracer activities^4^. In the latter study, the SUVmean in normal tissues was biased by less than 5% for both 10% and 30% activity levels, yet the 30% activity level was preferred, constituting a better tradeoff between reliability of SUV values and dose reduction^4^. Although very good performances with deep learning based methods are reported in recovering or denoising low-dose and ultra-low-dose PET images, those methods are currently time consuming in the training phase, and are not readily available in the clinical setting especially for brain imaging.

## Conclusion

Our simulation of an ultra-low dose protocol involving a 90% reduction of 18F-FDG injected activity did not significantly modify quantitative measurements of brain PET/MR images, suggesting that an activity as low as 0.2 MBq/Kg may be sufficient for reliable measurements of regional SUV ratios, and to differentiate AD from FTD. For visual interpretation, a substantial agreement was found between the standard (2 MBq/Kg) and ultra-low dose (0.2 MBq/Kg) protocols for patients with severe hypometabolism. The use of ultra-low doses would be of interest for younger patients, who may undergo repeated brain FDG-PET, for the diagnosis and monitoring encephalitis or psychiatric diseases, for example. It should be mentioned that reducing injected dose is of interest to improve the radiation protection of the PET technologists also. Reducing the injected dose also significantly reduces radiopharmaceutical costs in a department performing a lot of FDG-PET scans. However, for the early diagnosis of neurodegenerative disorders or when mild metabolism changes are expected, injecting 1–2 MBq/Kg (70–140 MBq) should be preferred to guarantee a precise diagnosis. The ultra-low-dose protocol offers a possibility of substantial reduction in ionizing radiation exposure, but should be reserved for the differential diagnosis or follow-up in patients with severe cognitive impairment or dementia.

## Data Availability

The data that support the findings of this study are available from Pitié-Salpêtrière Hospital but restrictions apply to the availability of these data, which were used under license for the current study, and so are not publicly available. Data are however available from the authors upon reasonable request and with permission of Pitié-Salpêtrière Hospital.

## Abbreviations

PET_ULD_: Positron emission tomography with ultra-low dose activity
PET_STD_: Positron emission tomography with standard activity
SUVR: Cortex-to-pons uptake ratios
SUVR_STD_: Cortex-to-pons uptake ratios for PET_STD_
SUVR_ULD_: Cortex-to-pons uptake ratios for PET_ULD_
N: Normal
NE: Non-evaluable
AD: Alzheimer’s disease
lvPPA: Logogenic variant of progressive primary aphasia
PCA: Posterior cortical atrophy
svPPA: Semantic variant of progressive primary aphasia
FTD: Frontotemporal dementia
CI: Confidence intervals
Ant.: Anterior
Post.: Posterior
Sup.: Superior
Inf.: Inferior
